# Assessing brain plasticity across the lifespan with transcranial magnetic stimulation: why, how, and what is the ultimate goal?

**DOI:** 10.3389/fnins.2013.00042

**Published:** 2013-04-02

**Authors:** Catarina Freitas, Faranak Farzan, Alvaro Pascual-Leone

**Affiliations:** ^1^Department of Neurology, Division of Cognitive Neurology, Berenson-Allen Center for Noninvasive Brain Stimulation, Beth Israel Deaconess Medical Center, Harvard Medical SchoolBoston, MA, USA; ^2^Institut Universitari de Neurorehabilitació Guttmann, Universidad Autónoma de BarcelonaBadalona, Spain

**Keywords:** brain plasticity, TMS, lifespan, aging, brain health index

## Abstract

Sustaining brain and cognitive function across the lifespan must be one of the main biomedical goals of the twenty-first century. We need to aim to *prevent* neuropsychiatric diseases and, thus, to identify and remediate brain and cognitive dysfunction *before* clinical symptoms manifest and disability develops. The brain undergoes a complex array of changes from developmental years into old age, putatively the underpinnings of changes in cognition and behavior throughout life. A functionally “normal” brain is a changing brain, a brain whose capacity and mechanisms of change are shifting appropriately from one time-point to another in a given individual's life. Therefore, assessing the mechanisms of brain plasticity across the lifespan is critical to gain insight into an individual's brain health. Indexing brain plasticity in humans is possible with transcranial magnetic stimulation (TMS), which, in combination with neuroimaging, provides a powerful tool for exploring local cortical and brain network plasticity. Here, we review investigations to date, summarize findings, and discuss some of the challenges that need to be solved to enhance the use of TMS measures of brain plasticity across all ages. Ultimately, TMS measures of plasticity can become the foundation for a *brain health index* (BHI) to enable objective correlates of an individual's brain health over time, assessment across diseases and disorders, and reliable evaluation of indicators of efficacy of future preventive and therapeutic interventions.

## Introduction: why assess brain plasticity across the lifespan

According to the World Health Organization, neuropsychiatric disorders affect one out of every five people over the course of their lives and represent the main cause of lifelong disability worldwide. The risk of neuropsychiatric disorders increases with age, and the world's population is rapidly growing and aging (Christensen et al., 2009). Thus, we face an expanding risk of neuropsychiatric age-associated disorders, including Alzheimer's disease (AD) (Alzheimer's Association, 2012), the impact of which cannot be overstated. Therefore, one of the most compelling biomedical goals of the twenty-first century must be to determine how to sustain brain and cognitive health across the lifespan aiming to *prevent* cognitive deterioration and neuropsychiatric diseases.

**Figure 1:**
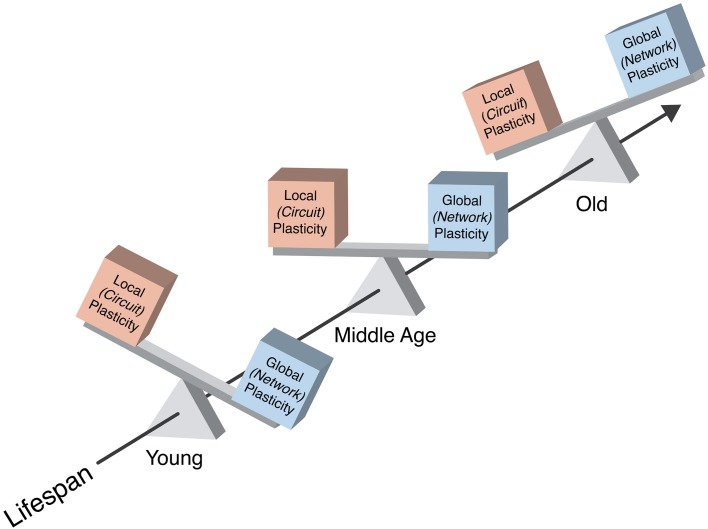
**Schematic representation of the balance between local and network plasticity and its change across a typical lifespan.** A functionally “normal” brain is a changing brain, a brain whose capacity and mechanisms of change are shifting appropriately from one time-point in life to another. In health, local cortical and network plasticity might keep a fine-tuned balance, which optimizes functionality.

The brain changes across the lifespan. First, growing evidence demonstrates that the brain undergoes a complex array of neuroanatomical and neurophysiologic modifications from birth till death, so that concepts such as “development” and “senescence” have become increasingly arbitrary in their definition. Instead, the lifespan and the aging process itself might be best viewed as a “life-long developmental process,” which is thought to constitute the underpinnings of shifts in cognition and behavior throughout each individual's life. Second, along with changes in brain structure and function, the mechanisms by which structure and function can be modified (the mechanisms of **brain plasticity** themselves) appear to also change over the lifespan. Such a “life-long dynamic, plastically changing brain” poses several challenges, including the definition of a functionally “normal” brain at a given point in time in a given individual. A functionally “normal” brain is a changing brain, a brain whose capacity and mechanisms of change are shifting appropriately from one time-point in life to another (Figure 1). Therefore, in evaluating an individual's brain health, we need a reliable and adaptable method to assess the mechanisms of brain plasticity across each individual's lifespan.

Different techniques and protocols have been developed and applied to assess neuroplasticity from infancy to late-life. We argue that **transcranial magnetic stimulation (TMS),** combined with other neurophysiologic or neuroimaging modalities, such as electromyography (EMG), electroencephalography (EEG), or functional magnetic resonance imaging (fMRI), is particularly useful for this purpose. Here, we review the studies on TMS measures of brain plasticity that have examined age-related changes to date. We aim not only to summarize findings and highlight the promise, but also to discuss some of the challenges yet to overcome in order to establish a reliable TMS measure of brain plasticity across all ages. We then propose an integrative approach incorporating TMS measures of plasticity whose ultimate goal should be the establishment of a ***brain health index*** (BHI). We believe that a reliable BHI, databased, and widely available for comparative purposes, would enable objective evaluation of an individual's brain health over time, assessment across diseases and disorders, guidance of strategies and interventions to sustain brain health and prevent problems, and reliable evaluation of indicators of efficacy of future preventive and therapeutic interventions.

## How to assess brain plasticity across the lifespan: transcranial magnetic stimulation

TMS is a versatile technique to non-invasively probe the human brain. TMS provides an outstanding tool to induce, measure, and modify local and network plasticity. TMS is safe if appropriate guidelines and precautions are followed (Rossi et al., 2009). TMS is based on electromagnetic induction and can be used to explore brain-behavior relations, map sensory, motor, and higher-order cognitive functions (Hallett, 2007), and examine the excitability, connectivity and plasticity of different cortical regions from newborns to the elderly (Pascual-Leone et al., 2011). Applied in trains of stimuli, repetitive TMS (rTMS) offers promise as a therapeutic intervention in a variety of nervous system diseases and disorders (Valero-Cabré et al., 2008). In this context, rTMS is applied to pre-selected, specific brain areas to modulate activity within and across functional networks that map onto symptoms of disease or disability (e.g., Strafella et al., 2001; for review, Shafi et al., 2012). To date, in the US, only the Neuronetics Neurostar TMS device and protocol are approved by the Food and Drug Administration for treatment of some patients with medication-resistant depression (O'Reardon et al., 2007; Connolly et al., 2012). However, other devices and protocols are being studied for other therapeutic applications of rTMS (e.g., Bentwich et al., 2011; Harel et al., 2011, 2012).

**Figure 2:**
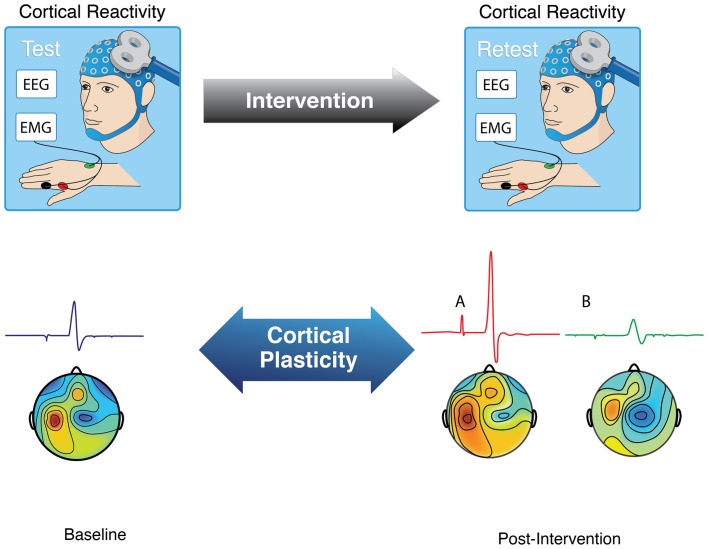
**Schematic representation of how to assess brain plasticity with TMS.** As recorded using either electromyography (EMG) or electroencephalography (EEG), brain responses to TMS can be measured as motor evoked potentials (when TMS is applied to motor cortex) or localized evoked field potentials. Comparison of these TMS-based measures of cortical reactivity before and after a given intervention (e.g., PAS, rTMS, TBS, task) can provide an index of brain plasticity in response to that intervention.

A number of experimental TMS measures of brain plasticity have been introduced. Fundamentally, single-pulse TMS combined with EMG, EEG, fMRI or other brain imaging methods can be used to quantify cortical reactivity before and following a given intervention (Figure 2). TMS can provide a controlled and quantifiable input that can be matched across individuals of different ages. Comparison of TMS measures of cortical reactivity before and after an intervention may thus provide an index of brain plasticity in response to said intervention. When the intervention itself involves TMS, it is possible to assess the efficacy of the mechanisms of plasticity in a defined cortical brain region (Figure 2). Here, we review paired-associative stimulation (PAS), and synchronous and asynchronous (theta-burst) rTMS techniques as interventions to induce plasticity and reliably quantify the efficacy of the underlying plasticity mechanisms.

### Paired-associative stimulation

PAS builds on the Hebbian principle of spike timing-dependent synaptic plasticity (Classen et al., 2004). In its most common form, PAS involves repeated pairing of median nerve electric stimulation with timed TMS over the contralateral primary motor cortex (e.g., 180 pairs delivered every 10 s). In this form, PAS has been shown to modulate the excitability of the motor system, leading to a lasting (≥60 min) increase in motor evoked potential (MEP) amplitudes when the interval between peripheral nerve stimulation and TMS is set at 25 ms (PAS25), an inter-stimulus interval (ISI) slightly longer than the time needed for the afferent inputs to reach the somatosensory cortex (N20 latency) (Stefan et al., 2000). Conversely, changing the interval between the two associative stimuli to 10 ms (PAS10)—i.e., an ISI shorter than the time needed for the afferent inputs to reach the cerebral cortex—leads to a depression of TMS-induced MEPs (Wolters et al., 2003).

The same principles of spike timing-dependency can be applied to other paradigms. For example, Cortes and colleagues introduced spinal associative stimulation (SAS) where a cortical stimulus is paired with a peripheral nerve stimulus such that the ISI results in confluence at the spinal level, thus promoting, when applied repetitively, segmental spinal plasticity (Cortes et al., 2011). A visual, auditory or other sensory stimulus can also be paired with a TMS stimulus to different brain regions in other versions of PAS (e.g., Wolters et al., 2005). Moreover, two TMS stimuli targeting different brain regions can be paired to evaluate and promote connectivity in specific neural networks.

### rTMS: Low- and high-frequency rTMS and theta-burst stimulation

rTMS consists in the application of a train of TMS pulses of the same intensity to a single brain area at a given frequency that can range from 1 to 20 or more stimuli per second. Such a train of rTMS can induce a modulation of cortical excitability beyond the duration of the train itself (Valero-Cabré et al., 2008). Depending on the stimulation parameters, particularly frequency and pattern of stimulation, cortical reactivity is potentiated or depressed. In general, a continuous train of lower frequencies of rTMS, in the 1-Hz range, leads to a transient suppression of excitability in the targeted cortical area, while bursts of high-frequency stimulation (≥5-Hz) lead to a temporary increase in cortical reactivity (Kobayashi and Pascual-Leone, 2003). However, it is important to realize that the relation between facilitatory and suppressive effects of rTMS trains of different parameters is not universal, and some combinations of rTMS frequency and intensity, or the state of brain activity at the time of rTMS, may result in paradoxically opposite effects than expected. As an example of the former, intermittent 6-Hz rTMS applied at sub-threshold intensity (i.e., below motor threshold) was shown to suppress motor cortical excitability for ~30 min after the end of stimulation (Todd et al., 2006). Examples of the latter have also been provided (e.g., Silvanto et al., 2008; Weisz et al., 2012; for review, Silvanto and Pascual-Leone, 2008).

The modulatory effects of theta-burst stimulation (TBS) on local cortical reactivity aim to mimic paradigms used to assess synaptic plasticity in animal models (Huang et al., 2005, 2008). Specifically, TBS involves application of 3 bursts of 50-Hz rTMS repeated every 200 milliseconds either continuously for a total of 40 s or intermittently (every 8 s) for about 3 min. When applied to the motor cortex, continuous (cTBS) and intermittent TBS (iTBS) were shown to result in depression and potentiation of cortical reactivity as indexed through suppression and facilitation of MEPs, respectively (Huang et al., 2005). Despite the relatively short duration of TBS (<4 min) compared to conventional rTMS (~20 min), the alteration of cortical excitability by TBS can last for about 70 min, which is more than twice as long as the conventional rTMS approaches (Thut and Pascual-Leone, 2010; Di Lazzaro et al., 2011).

Hoogendam et al. (2010) argue that several lines of evidence strongly suggest a link between the after-effects induced by rTMS and induction of synaptic plasticity (e.g., rTMS has effects that outlast the period of stimulation, the temporal pattern of pulses, changes in excitability induced by rTMS depend on the history of activation, pharmacologic experiments suggest dependency on glutamatergic mechanisms, rTMS interacts with learning, etc.). Results of animal and human studies are consistent with the notion that the modulatory effects of TBS on cortical reactivity reflect long-term potentiation (LTP)- and long-term depression (LTD)-like mechanisms (for review, Cárdenas-Morales et al., 2010). However, the mechanisms of action of rTMS, including TBS, remain insufficiently understood. Indeed, the cellular actions of rTMS and TBS likely encompass multiple interacting phenomena (Reithler et al., 2011), including changes in excitatory synaptic transmission (Huang et al., 2011), modulation of inhibitory cortical activity (Funke and Benali, 2010), shifts in membrane potentials (Pell et al., 2011), modulation of distributed network activity (Shafi et al., 2012), and stochastic resonance (Schwarzkopf et al., 2011). Further mechanistic studies are certainly needed, yet results to date illustrate the utility of TMS measures in the assessment of brain plasticity across the lifespan.

### Why use TMS to assess brain plasticity

We argue that using TMS as intervention (PAS, rTMS, or TBS) to evaluate brain plasticity across the lifespan offers multi-fold advantages. First, from a purely electro-mechanical system perspective, TMS represents a controlled input to the brain's system that can be matched across brain regions and individuals (regardless of age) and allow demodulation of the TMS-related input. By controlling the input to the brain, it is possible to quantify local response and the dynamic spatial spreading pattern and propagation speed of the induced activity. By comparing these spatial parameters in young and older adults, for instance, it may be possible to not only assess mechanisms of local plasticity, but also quantitatively test the processing speed deficit hypothesis in the aging brain and map spatial characteristics of neural activity spreading to anatomical features identified in structural MRI.

Precise targeting of a specific cortical region can be accomplished using individual brain MRI-guided neuronavigation (discussed in more detail in section “Neuronavigated and Multimodal TMS”). Single-pulse TMS can be applied and local cortical responses recorded by EMG (when testing motor cortex) or EEG as TMS-evoked potentials (TEP, when testing any cortical, including non-motor, areas). Global field power (GFP) of the TEP can be used as primary outcome and parametric variation of TMS intensity enables definition of an input–output relation as a metric of cortical reactivity. Paired-pulse TMS with variable ISIs allows exploration of intra-cortical inhibitory and facilitatory interactions, which pharmacologic studies suggest are related to GABA_A_ and glutamatergic mechanisms. To exemplify, comparison of such measures of single and paired-pulse TMS between young and older individuals will provide novel insights onto age-related changes in cortical reactivity.

On the other hand, if, as discussed in section “Challenges and Advancements to Use TMS Measures of Plasticity Across the Lifespan,” several important factors are appropriately considered and controlled for, comparison of cortical reactivity before to after PAS, conventional rTMS, or TBS can provide a reliable assessment of cortical plasticity mechanisms. In aging studies, for example, such measures of TMS can be applied longitudinally across different brain regions within individuals to assess age-related modification of cortical plasticity. Furthermore, rTMS and PAS can be applied to different cortical regions, such as primary motor cortex *vs.* dorsolateral prefrontal cortex (Rajji et al., 2011), and single-pulse TMS in combination with EEG recording can be used before and after such interventions to evaluate cortical plasticity in each of these brain regions. In lifespan studies, comparison of cortical plasticity between brain regions will provide further insight onto the selective effect of aging on specific brain regions and networks.

## Assessing brain plasticity across the lifespan: TMS studies to date

Much can be learnt from studying brain plasticity mechanisms with a large number of different approaches (Pascual-Leone et al., 2005). For example, a number of studies have examined age-related changes in use-dependent plasticity where the intervention (Figure 2) was a motor task. To date, results from such studies have been inconsistent and inconclusive, perhaps illustrating the challenges of applying behavioral interventions to assess plasticity across the lifespan. For instance, Rogasch et al. (2009) had subjects perform a task that involved maximizing peak thumb abduction acceleration during ballistic movements of the right thumb. Although peak thumb acceleration was similar between younger and older individuals at the beginning of the training, it became significantly greater by the end of the training in the young. In this context, the authors found differences in corticomotor plasticity between young and elders. However, given the differences in motor training and task performance, the interpretation of such findings is difficult. Cirillo et al. (2011) used a more complex motor task (visuomotor tracking) and found no difference in corticomotor plasticity between younger and older adults. Although the extent of motor learning—quantified by improvement in visuomotor tracking error—was similar between groups, visuomotor tracking performance was diminished in the elderly as compared to the younger. Again, behavioral differences make interpretation of the measures of plasticity challenging. Nevertheless, major findings of all four articles published thus far on use-dependent plasticity in healthy younger and elderly individuals (Rogasch et al., 2009; Cirillo et al., 2010, 2011) or across the age-span (Sawaki et al., 2003) are presented in Table 1.

**Table 1:** **Studies of induction of brain plasticity using or assessed by TMS in healthy subjects across the lifespan**.

**Paper**	***N***	**Age (yrs)**^**¶**^	**Gender (% M)**	**Type of study**	**NeuroΨ testing**	**Outcome measure**	**Main findings**	**Comments**
**THETA-BURST STIMULATION (TBS)**								
Freitas et al., 2011	36	50.3 ± 18.5 (age-range: 19–81 yrs)		Single design (cross-sectional study)	EHQ MMSE (≥29)	MEP (FDI)	cTBS-induced LTD-like plasticity progressively diminished with advancing age	Significant correlations between time-to-baseline/MEP area/minimum MEP amplitude with age
**CONVENTIONAL REPETITIVE TRANSCRANIAL MAGNETIC STIMULATION (rTMS)**								
Todd et al., 2010	30	15 young: 25.0 ± 4.0	Young: 60%	Randomized, double-blind, parallel design (cohort study)	EHQ (TMS safety screen)	MEP (FDI)	Inhibitory rTMS-induced LTD-like plasticity reduced in the elderly	MEP area and amplitude reduced in younger but not in older; no effect of gender
		15 elderly: 67.0 ± 5.0	Older: 60%					
**PAIRED ASSOCIATIVE STIMULATION (PAS)**								
Fathi et al., 2010	48	16 young (21–39 yrs)	Young: 87.5%	Parallel design (cohort study)		MEP (APB)	PAS_25_-induced LTP-like changes obtained in young and middle-aged but not in the elderly	No effect of gender; mean peak N20 latency in the elderly was within normal limits
		16 middle (40–59 yrs)	Middle: 43.8%					
		16 elderly (60–79 yrs)	Older: 68.8%					
Pellicciari et al., 2009	32	16 young: ^*^26.2 ± 0.8	Young: 50%	Parallel design (cohort study)		SEP	PAS_*N*20_-induced LTP-like changes obtained in both groups but significantly higher the in elderly group	SEP measured by N20-P25 complex; all subjects studied within same period of the day; young females were in follicular phase and older females in menopause
		16 elderly: ^*^62.1 ± 1.5	Older: 50%					
Müller-Dahlhaus et al., 2008	27	38.2 ± 3.1 (age-range: 22–71 yrs)	40.7%	Single design (cross-sectional study)		MEP (APB)	PAS_*N*20+2_-induced LTP- and LTD-like changes decreased in the elderly	Substantial inter-individual variability
Tecchio et al., 2008	50	25 young: 29.8 ± 4.5	Young: 48%	Parallel design (cohort study)	EHQ	MEP (APB)	PAS_25_-induced LTP-like plasticity reduced only in older women (not in older men)	*t*-PMP (PAS-induced MEP potentiation estimate) was used for data analysis
		25 elderly: 61.1 ± 4.1	Older: 48%					
**TRAINING-DEPENDENT PLASTICITY (TDP)**								
Cirillo et al., 2011	32	16 young: 23.0 ± 3.0	Young: 43.8%	Parallel design (cohort study)	EHQ MMSE IPAQ	MEP (FDI^†^, ADM) SICI	No difference in corticomotor plasticity (or SICI) after visuo-motor tracking between young and old	Task performance diminished in older compared with younger but extent of motor learning did not differ between groups
		16 elderly: 67.0 ± 5.0	Elderly: 43.8%					
Cirillo et al., 2010	26	12 young: 22.0 ± 2.0	Young: 41.7%	Parallel design (cohort study)	EHQ IPAQ	MEP (APB^†^, ADM) SICI	No difference in corticomotor plasticity after repetitive thumb abduction between young and old	Age-related decline in motor learning only in the dominant hand. Unchanged SICI in either hand in both groups after training
		14 elderly: 61.1 ± 4.1	Elderly: 50%					
Rogasch et al., 2009	28	14 young: 20.7 ± 1.9	Young: 57.1%	Parallel design (cohort study)	EHQ	MEP (APB^†^, ADM) SICI	No difference in corticomotor plasticity after peak thumb abduction acceleration training in older but significantly enhanced in younger	Improvement in task-specific ballistic motor performance diminished in older. Unchanged SICI in both groups after training
		14 elderly: 68.3 ± 6.5	Elderly: 57.1%					
Sawaki et al., 2003	55	44.0 ± 16.0 (age-range: 18–85 yrs)	50.9%	Single design (cross-sectional study)		MEP (EPB, FPB)	Magnitude of corticomotor plasticity after simple thumb movement task inversely correlated with age	Changes could not be accounted for by motor training kinematics

LTP, Long-term potentiation; LTD, Long-term depression; cTBS, Continuous theta-burst stimulation; MEP, Motor evoked potential; SEP, Somatosensory evoked potential; SICI, Short-interval intra-cortical inhibition; PAS_25_, Paired associative stimulation applied at an inter-stimulus interval (ISI) of 25ms; PAS_N20+2_, Paired associative stimulation applied at an ISI of the individual N20 latency of the median nerve SEP plus 2 ms; PAS_N20_, Paired associative stimulation applied at an ISI of 20 ms (N20 latency); ADM, Abductor digiti minimi muscle; APB, Abductor pollicis brevis muscle; EPB, Extensor pollicis brevis muscle; FDI, First dorsal interosseous muscle; FPB, Flexor pollicis brevis muscle; EHQ, Edinburgh Handedness Questionnaire; IPAQ, International Physical Activity Questionnaire; MMSE, Mini-mental State Examination; Yrs, Years; M, Male;

¶ Values presented as mean ± standard deviation;

* Mean ± standard error;

† Target muscle.

Herein, we review all peer-reviewed studies that have used TMS as the intervention and investigated TMS-driven measures of plasticity to characterize neuroplastic phenomena throughout adult life. We separate studies into *cohort* studies, which compare two, somewhat arbitrarily defined, groups of healthy subjects (i.e., “younger” *vs.* “older”) from *cross-sectional, lifespan* studies, which examine the effect of the age *spectrum* on TMS measures of plasticity (generally applying correlation analysis). Main characteristics and findings of all studies in which brain plasticity was experimentally induced by various TMS protocols are presented in Table 1.

### Cohort TMS plasticity studies

Several investigations have used cohorts of healthy individuals of different age ranges and compared TMS measures of plasticity in young *vs.* older adults. Todd et al. (2010) applied rTMS whilst others utilized PAS (Tecchio et al., 2008; Pellicciari et al., 2009; Fathi et al., 2010). Overall, these studies have shown reductions in plasticity (both LTP- as well as LTD-like plasticity) with physiological aging in the motor cortex, and enhancement of LTP-like plasticity in the somatosensory cortex, potentially as a compensatory mechanism.

For the most part, PAS studies have shown differences in LTP-like plasticity in healthy older compared to younger subjects. Tecchio et al. (2008) found a reduction in PAS(25)-induced plasticity in older women, but not in older men. Fathi et al. (2010) obtained PAS-induced LTP-like changes in young and middle-aged but not in the elderly, with no effect of gender. On the other hand, and contrasting to the apparently diminished motor cortical plasticity, somatosensory plasticity—induced by a PAS paradigm in which TMS is applied to the contralateral primary somatosensory cortex (Wolters et al., 2005)—appears to be enhanced in older adults. Pellicciari et al. (2009) found that PAS intervention resulted in larger amplitudes of the N20-P25 complex of somatosensory-evoked potentials (SEP) in older than in younger healthy individuals. It has been suggested that N20 and P25 reflect the initial excitation of neurons in areas 3b and 1, which can be significantly affected by aging (Allison et al., 1984, but see Pellicciari et al., 2009).

In a sham-controlled experiment using high-frequency, low-intensity rTMS (Todd et al., 2006), Todd et al. (2010) demonstrated that older adults appear to exhibit diminished corticomotor LTD-like plasticity compared to younger healthy subjects.

### Cross-sectional, lifespan TMS plasticity studies

**Figure 3:**
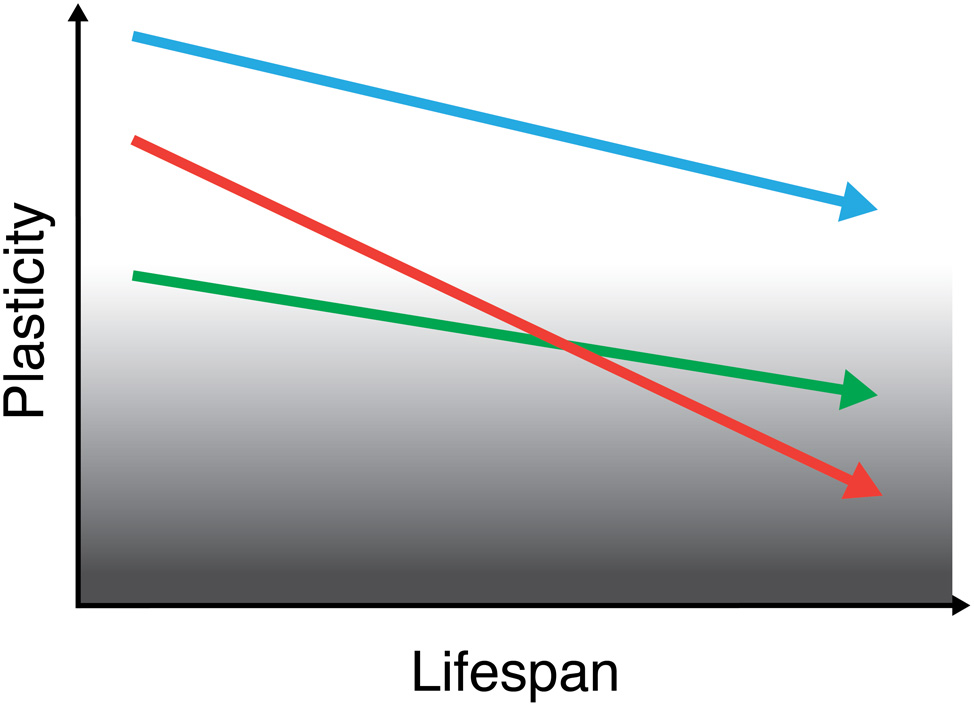
**Schematic representation of brain plasticity across the lifespan.** Brain plasticity progressively declines throughout the (adult) lifespan, putatively underlying a decline in cognitive function. Although mechanisms of plasticity show a downward trend over the course of a typical lifetime, this trend will manifest differently according to initial “baseline” levels, genetic factors, and environmental influences. Therefore, each individual may have a unique “slope of plasticity” across the lifespan. Assessing the trajectories of brain plasticity across each individual's lifespan may shed light into how the brain continues to sustain healthy functionality throughout life in some individuals and how functionality is impaired, ultimately leading to the manifestation of brain disease, in others. Lines in figure intend to depict the life-course of three different individuals.

Cross-sectional, lifespan investigations to date seem to complement the aforementioned cohort studies and suggest that reduction in plastic efficiency may constitute a continuous, gradual, and insidious declining process taking place throughout the adult age *spectrum* (Figure 3). Lifespan studies may, therefore, provide additional and important information to characterize and better understand the brain's adaptive (or compensatory) mechanisms associated with aging.

Two studies have examined experimentally-induced plasticity throughout adult lifespan. Based on the hypothesis that plasticity mechanisms may become increasingly less efficient across the lifespan, in Freitas et al. (2011) we applied cTBS to assess LTD-like phenomena. By studying a group of 36 healthy individuals ranging in age from 19 to 81 years, we demonstrated that there is a steady and progressive decline of the efficiency of the corticomotor mechanisms of plasticity with advancing age. On the other hand, employing PAS, Müller-Dahlhaus et al. (2008) studied a group of 27 healthy individuals ranging in age from 22 to 71 years. PAS was applied at an ISI corresponding to each individual's N20 latency of the median nerve cortical SEP plus 2 ms (PAS_N20+2_), which induced the expected LTP-like changes in about half (52%) the subjects while it induced LTD-like plasticity in the remaining (48%). Irrespective of the direction, however, the magnitude of the absolute PAS_N20+2_ effect decreased linearly as a function of age (Müller-Dahlhaus et al., 2008).

Certainly, such cross-sectional studies are promising, but more work is needed. Neuroimaging modalities, which will be discussed later, can offer valuable insights into network plasticity and the nature and extent of interaction between cortical areas. Additionally, further work would also assist in fully understanding how such intrinsic changes are influenced by genetic as well as environmental factors, and how those changes are modulated and evolve throughout the age-span (Pascual-Leone and Taylor, 2011).

## Challenges and advancements to use TMS measures of plasticity across the lifespan

### Morphometry, TMS parameter optimization, genotyping

TMS protocols that have an effect in the normal young brain may not have the same effect in the aged brain (Zimerman and Hummel, 2010). The following paragraphs briefly discuss a few factors we consider most relevant among a plethora of variables that can influence findings and need to be carefully controlled when applying TMS measures to assess brain plasticity across the lifespan.

#### Systematic morphometric analysis

Morphometric brain changes associated with healthy aging, including regional cortical thinning, are well-established and consistently demonstrated in either cross-sectional (e.g., Walhovd et al., 2005, 2011; Fjell et al., 2009) or longitudinal studies (e.g., Raz et al., 2005; Driscoll et al., 2009). Such age-related brain atrophy implies an increase of TMS coil-to-hotspot distance that is relevant, since TMS effects depend on the distance between cortex and scalp, and the magnetic field (i.e., the induced current in the brain) decreases with distance (Wagner et al., 2004, 2008). Furthermore, brain atrophy can substantially alter the effect of TMS not only because of greater scalp-to-brain distance but also due to increased current shunting in the cerebrospinal fluid (CSF) compartment (Wagner et al., 2007). Modeling work suggests that current density distribution is critically influenced by both brain morphology and tissue characteristics (Wagner et al., 2007, 2008). Therefore, it is conceivable that the differences in TMS effects in healthy young *vs.* elderly might be highly biased if brain atrophy is not accounted for. Volumetric studies of cortical thinning, white matter density, and the CSF layer should be included in future studies to assist in the interpretation of TMS results involving subjects of all ages.

#### Optimization of TMS parameters

Induction of plastic changes in the elder brain with TMS may require different parameters from those used to induce plastic changes in the younger brain. That is, TMS parameters might need to be modified in order to achieve comparable effects on cortical reactivity in older and younger subjects. For instance, in their experiment on age and gender differences in motor cortical input–output characteristics, Pitcher et al. (2003) found that higher stimulus intensities were required to achieve TMS-induced MEP_max_ (maximal amplitude of the MEP that can be evoked) in older as compared to younger subjects—i.e., greater stimulus intensities were required to reach the same maximal motor output in older subjects—and yet the amplitude of the MEP_max_ remained unaffected by age. This suggests that the amount of the reported plastic changes might be partly impacted by the intensity of the TMS intervention.

Moreover, the effects of TMS depend critically on stimulation frequency and type of protocol used to probe plasticity. There is, in fact, substantial inter- and even intra-individual variability in the modulation of corticospinal excitability by rTMS (Maeda et al., 2000), even at different frequencies (1-, 10-, 15-, and 20-Hz). Similarly, the after-effects of continuous and intermittent TBS over the motor cortex were recently shown to be highly variable between individuals, likely due to being strongly influenced by which interneuron networks are recruited by the TMS pulses (Hamada et al., 2012). Nevertheless, neuromodulation through TBS might be more consistent within and across subjects than with more conventional rTMS protocols; in turn, PAS-induced neuromodulation might be approximately as robust as TBS-induced modulatory effects. Indeed, Di Lazzaro et al. (2011) compared the after-effects of six different TMS protocols [PAS(25) and PAS(10); cTBS and iTBS; 1- and 5-Hz rTMS] on the excitability of the stimulated and contralateral motor cortex in healthy subjects. They found that a pronounced increase of cortical excitability—evaluated by measuring the amplitude of MEPs—was produced by iTBS (+56%) and PAS(25) (+45%), whereas 5-Hz rTMS did not produce a significant increase of MEPs. On the other hand, a pronounced decrease of cortical excitability was produced by PAS(10) (−31%) and cTBS (−29%), and, to a lesser extent, by 1-Hz rTMS (−20%) (Di Lazzaro et al., 2011). However, Player et al. (2012) found that PAS(25) increases motor cortex excitability more effectively than iTBS. These results demonstrate that the efficacy of various TMS paradigms might differ and the impact of age on such differences remains unclear. Therefore, further investigations are warranted to evaluate in more detail which TMS protocol is able to induce the most consistent (and more meaningful) modulatory effects in individuals of different ages.

#### Genotyping

So far, few but salient investigations have explored the impact of single nucleotide polymorphisms (SPN) and their interaction on TMS measures of cortical plasticity. For instance, Witte et al. (2012) evaluated the effects of the common SPN brain-derived neurotrophic factor (BDNF) valine-to-methionine substitution at codon 66 (Val66Met) genotype—which is found in ~33% of the Caucasian population (Egan et al., 2003)—on PAS(25)-induced motor cortex plasticity. The authors also considered catechol-O-methyltransferase (COMT) Val158Met and kidney and brain (KIBRA) rs17070145 carrier status. They found that while BDNF carrier status alone did not significantly influence PAS-induced cortical plasticity, a significant BDNF × COMT interaction was present such that the BDNF Val/Val *vs.* Met genotype in COMT Met homozygotes showed higher plasticity immediately following the PAS(25) protocol (Witte et al., 2012).

In a comprehensive study of the influence of BDNF Val66Met on various TMS probes of plasticity, Cheeran et al. (2008) found that the response of Met allele carriers differed significantly in all protocols compared with the response of Val66Val individuals. Specifically, (1) induction of LTP/LTD-like plasticity resulted in a significant time × genotype interaction for both iTBS and cTBS, with a significant increase in MEPs after iTBS and a significant decrease in MEPs after cTBS in the Val/Val individuals but not in the non-Val/Val group; (2) control of homeostatic plasticity—in which sub-threshold 1-Hz rTMS was pre-conditioned by cathodal transcranial direct current stimulation (tDCS) to generate facilitation of the motor cortex and thus produce a homeostatic-like effect (Siebner et al., 2004)—resulted in a significant time × genotype interaction, with significantly higher MEP amplitudes after 1-Hz rTMS in the Val/Val group compared to the non-Val/Val group; and (3) PAS(25) for two muscles [abductor pollicis brevis (APB); abductor digiti minimi (ADM)] resulted in a significant increase of the MEPs in ADM and a borderline significant increase in APB in the Val/Val group and no significant effects in non-Val/Val individuals (Cheeran et al., 2008).

In the same vein, Jayasekeran et al. (2011) demonstrated that the BDNF Val66Met polymorphism affects plasticity of the pharyngeal motor cortex to different forms of neurostimulation: after 5-Hz rTMS, there was a significant reduction of MEP latencies in subjects with the SPN that encoded Met66; moreover, the expected inhibitory effect of 1-Hz rTMS on MEP amplitude was not observed in carriers of the BDNF Val66Met polymorphism. Mori et al. (2011) showed that genetic variants of the NMDA receptor influence cortical excitability (as assessed by single- and paired-pulse TMS) and LTP-like plasticity (induced by iTBS) in healthy subjects.

Therefore, future studies should consider genetic factors and various polymorphisms that might impact TMS plasticity measures. Indeed, genetic factors clearly influence the effects of TMS (Hoogendam et al., 2010), and age-related changes in gene expression profile might play a role in age-related differences and the suitability of different TMS paradigms to optimally assess plasticity in individuals of different ages (as discussed above).

### Neuronavigated and multimodal TMS

We have used neuronavigated TMS and morphometric MRI in a study on TMS measures of plasticity in a small subset of subjects ranging in age from 20 to 73 years (Freitas et al., 2011). Further and larger studies employing neuronavigation and multimodal TMS techniques are needed.

#### Navigated TMS

**Figure 4:**
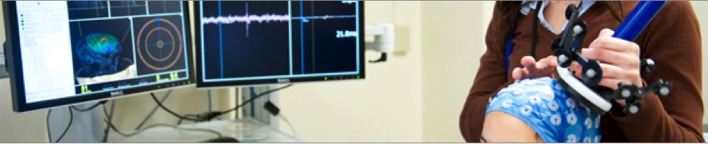
**Illustration of multimodal, neuronavigated TMS**.

The precise and accurate positioning of the TMS coil can be addressed by modern neuronavigation strategies (Gugino et al., 2001; Sparing et al., 2010) and enhanced precision of coil placement minimizes inter- and intra-subject variability of the effects of TMS (Sack et al., 2009). Neuronavigation can be used in real time throughout the experiment to guide and monitor the location of the TMS coil in relation to the subject's head on an individual basis (Herwig et al., 2001) (Figure 4). Ruohonen and Karhu (2010) have recently reviewed and highlighted the physics and physiology behind the accuracy and reproducibility of navigated TMS, among other topics of similar interest. In regard to TMS measures of plasticity, by comparing navigated to non-navigated TMS over the motor cortex, Julkunen et al. (2009) showed that the stimulus location was more spatially discrete in navigated TMS and produced more stable MEPs with significantly higher amplitudes and shorter latencies. In other words, the investigators showed that whilst the motor thresholds were not significantly dependent on the discrete stimulation site, MEPs exhibited significant differences depending on whether navigation is used. Bashir et al. (2011) demonstrated that neuronavigation increases the physiologic and behavioral effects of low-frequency rTMS over the primary motor cortex in healthy subjects; moreover, navigated TMS also resulted in a more robust modulation of the contralateral (unstimulated) hemisphere. Thus, it appears that navigated TMS is critical to maximize the reliability of the TMS intervention in the proposed studies of TMS measures of plasticity.

#### Multimodal TMS

In addition to the utility of neuronavigation for planning, monitoring, and documenting the location of the TMS coil relative to the subject's brain, novel methods based on the combination of TMS with neuroimaging technologies allow the assessment of excitability, plasticity, and connectivity of the human brain (Paus, 1999). Comprehensive reviews on this topic have been recently published (e.g., Wagner et al., 2007; Thut and Pascual-Leone, 2010; Reithler et al., 2011; Shafi et al., 2012).

Navigated TMS can thus be complemented with multimodal technologies such as EEG, fMRI, and positron emission tomography (PET) (Figure 4). Neuroimaging has indeed gone through a number of advancements in the past few years, and the combination of TMS and brain imaging techniques can enhance the reliability and validity of TMS indices of plasticity. Specifically, (1) combining TMS with MRI enhances TMS reliability in relation to targeting resolution and coil placement; (2) TMS combined with EEG enhances temporal resolution and allows the study of network plasticity as well as state-dependency; and (3) TMS combined with fMRI enhances spatial resolution and allows the exploration of age-related changes in non-cortical structures.

### Expansion of TMS test-retest reliability studies

#### Reliability of TMS-related measures/multimodal TMS methods

Thorough testing of the reliability and validity of TMS measures and multimodal TMS methods is critical. To date, important studies in healthy and diseased populations were able to demonstrate good test-retest reliability for several TMS-related measures in motor (Carroll et al., 2001; De Gennaro et al., 2003; Humm et al., 2004; Malcolm et al., 2006; Paine et al., 2006; Christie et al., 2007; Koski et al., 2007; Plowman-Prine et al., 2008; Doeltgen et al., 2009; Lioumis et al., 2009; Wheaton et al., 2009; Farzan et al., 2010; Cacchio et al., 2011; Badawy et al., 2012; Ngomo et al., 2012) and prefrontal cortices (Lioumis et al., 2009; Farzan et al., 2010). In visual cortex, TMS measures appear to show high test-retest reliability for phosphenes but not for suppression of visual perception (Siniatchkin et al., 2011).

Test-retest reliability of TMS for human motor cortex mapping has been demonstrated (Mortifee et al., 1994; McMillan et al., 1998; Corneal et al., 2005; Malcolm et al., 2006; Plowman-Prine et al., 2008; Hetu et al., 2011). In fact, measures of test-retest reliability might be valuable indicators of age-related brain changes (McGregor et al., 2012). Reliability of the TMS-EEG method has been supported by Lioumis et al. (2009), who showed a high overall reproducibility in the TMS-EEG responses over both hemispheres for both motor and prefrontal cortical stimulation, as well as by Casarotto et al. (2010), who demonstrated that EEG responses to TMS are sensitive to changes in the TMS perturbation parameters and repeatable over time.

Nevertheless, test-retest (or repeatability) studies assessing the stability and reliability of TMS measures of plasticity and multimodal TMS methods are still sparse and need expansion. To date, only a couple of studies have assessed the reliability of PAS, conventional rTMS, and TBS, and results remain somewhat unclear and limited. Fratello et al. (2006) showed that LTP-like plasticity induced by PAS(25) caused a reproducible increase in MEP amplitude, but failed to find acceptable intra-individual reliability. Martin et al. (2006) showed that the magnitude and reliability of cTBS depends on the targeted cortical region (MEPs were significantly depressed 5 min after TBS over FDI, but highly variable when targeting biceps), which points to the possibility that the expected stimulation after-effects may not be similar across all brain regions.

#### Accounting for state-dependency and other factors in the context of test-retest studies

Reliability studies should account for state-dependent effects, i.e., the state of neuronal activation in the targeted brain region at the time of stimulation, as a growing number of studies indicate that TMS effectiveness strongly depends on it. Moreover, state-dependency may also change over the lifespan. For an in-depth review and insights into how the systematic study of state-dependency can enhance the effectiveness of TMS in investigations on the neural basis of perception and cognition, see Silvanto and Pascual-Leone (2008). Importantly, a TMS measure can be reliable even though it is state-dependent as long as the state-dependency is accounted for, and the state is monitored. As pinpointed by Rossi et al. (2009), several variables (or a combination of them) may contribute to change the pre-TMS level of neuronal activity—thereby changing the resulting TMS effects (and risks)—and may, thus, critically contribute to the differences between healthy male and female participants, between patients with various diseases, and even across individuals and within individuals over time. Other variables that might contribute to change test-retest reliability involving TMS, by influencing the basal level of neuronal activity, may include time of day (Sale et al., 2007), hormonal changes (e.g., menstrual cycle, post-menopause), level of anxiety or mood, sleep deprivation, or occult substance abuse (Rossi et al., 2009). For instance, Cohen et al. (2010) found that TMS measures of plasticity can show significant diurnal changes, consistent with chronobiologic factors. Therefore, careful consideration of such factors in test-retest reliability studies is critical.

## The ultimate goal: a “brain health index”

The integrity of the neurophysiologic mechanisms underlying brain plasticity plays an important role throughout the lifespan in health and also in disease. In health, local cortical and network plasticity might keep a fine-tuned balance (Figure 1), which optimizes functionality (Pascual-Leone et al., 2011). Longitudinal studies of neurophysiologic plastic phenomena in the healthy population and, in particular, of the dynamics of local cortical and network plasticity mechanisms—*in vivo* and as assessed by TMS—are, to date, lacking. Whereas there is preliminary evidence from cross-sectional (and, indirectly, also from cohort) studies that a progressive dampening in the efficiency of plasticity mechanisms may occur across the adult lifespan, longitudinal studies of brain plasticity would be of crucial importance to understand in greater depth how the brain continues to sustain healthy functionality throughout life in some individuals and how functionality is impaired, ultimately leading to the manifestation of brain disease, in others (Figure 3).

The construct of cognitive reserve (Stern, 2009) is highly pertinent for this context. However, even though cognitive reserve is intimately related to cortical plasticity, it remains poorly understood at the present time (for review, Esiri and Chance, 2012). Cognitive reserve seemingly allows cognitive function to be maintained—or minimally disrupted—in older age and can enable individuals to sustain a greater amount of neuropathological insults before they manifest signs and symptoms of cognitive decline, and cognition-related disorders and dementia. A number of lifestyle factors including education, work complexity, social network, and leisure activities seem to contribute to this reserve (Scarmeas and Stern, 2003; Fratiglioni and Wang, 2007). For instance, highly educated individuals, even those with neuropathologic AD (amyloid burden), seem to have increased resistance to dementia (Roe et al., 2007). In mild AD patients with the same degree of cognitive deterioration, highly educated patients have more advanced pathological and functional brain changes (Kemppainen et al., 2008), which suggest that the clinical manifestation of advanced AD pathology is delayed in individuals with higher educational attainment (Stern et al., 1992; Alexander et al., 1997). In the same line of evidence, Landau et al. (2012a) reported a direct association between lifetime cognitive engagement and amyloid burden, inasmuch as greater participation in cognitively stimulating activities across the lifespan, particularly in early and middle life, was associated with reduced amyloid burden; moreover, older participants in the highest cognitive activity tertile had β-amyloid deposition comparable to young controls, whereas those in the lowest cognitive activity tertile had β-amyloid deposition comparable to patients with AD, thus suggesting that certain lifestyle factors, such as high cognitive engagement, may prevent or slow deposition of β-amyloid (Landau et al., 2012a). Hypothetically, this may be due to more efficient, compensatory, plasticity-based mechanisms against the underlying pathology. Adaptive (or compensatory) network plasticity might, thus, represent the neurobiological substrate of cognitive reserve, but much more work is required to actually characterize and understand the mechanisms involved.

**Figure 5:**
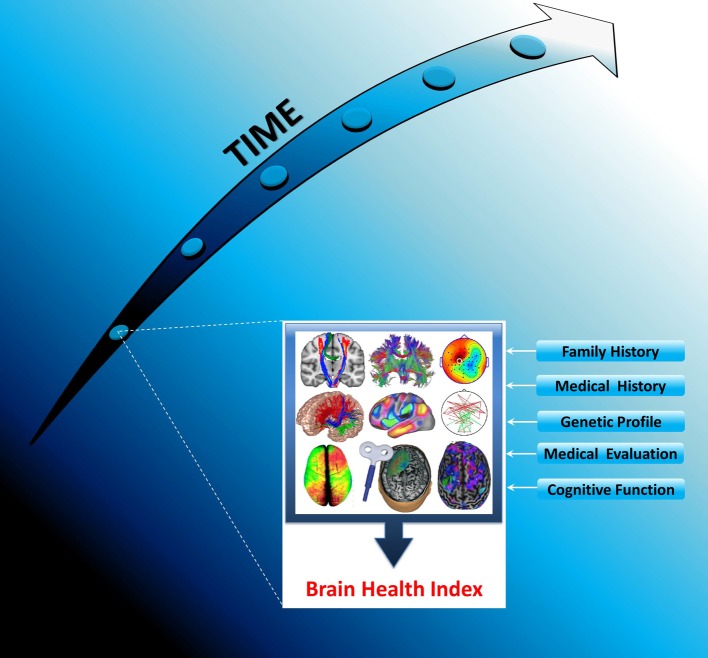
**Establishing a *Brain Health Index* (BHI): Schematic representation of the longitudinal monitoring of each individual's BHI across the lifespan.** The BHI includes neuronavigated, multimodal TMS measures suitable for identifying complex interactions between genes and environmental factors. Longitudinal monitoring of each individual's BHI would allow the comparison of individual's brain plasticity and network dynamics at every point in time to the previous history of that individual, thereby making it possible to identify pathological changes prior to manifestation of neuropsychiatric symptoms. To achieve this, the data gathered at the initial assessment and each follow-up, for each individual, will be added to a database to allow researchers to identify individual biomarkers for risk of disease with the ultimate goal of offering individualized, preventive interventions, and further monitor disease progression and treatment response.

*In vivo* longitudinal plasticity studies of normal human aging would, thereby, be highly relevant to understand in greater depth sustenance of healthy brain and cognitive function across the lifespan and appearance of brain disease. Furthermore, the need for longitudinal studies of normal aging is emphasized by their potential to detect very early, “outlier,” potentially pathologic changes. On the other hand, plastic changes may be further induced by a panoply of genetic, lifestyle, and environmental factors, which may trigger a cascade of events potentially leading to cognitive deterioration (and even dementia) in the absence of successful local and network compensatory strategies. Therefore, longitudinally assessing how such factors operate and interact with neuroplasticity is most needed. In addition, the study of the link between plasticity measures assessed longitudinally with measures of cognition and behavior would allow for a much more integral understanding of brain-behavior interactions. Finally, the relation between such longitudinally studied measures of plasticity and biomarkers such as amyloid deposition (Landau et al., 2012b) or CSF levels of β-amyloid (Aβ_42_) (van Harten et al., 2012) or of interleukin-12 (IL-12) and IL-23 subunit p40 (vom Berg et al., 2012) might enable the prediction of the development of disorders of cognition and behavior, most especially AD. In essence, we propose an integrative approach in which the longitudinal study (with predefined interim assessments) of TMS plasticity measures is correlated with comprehensive assessments of cognition and behavior, other brain markers of pathogenesis, and genetic and lifestyle factors with the ultimate goal of seeking to establish an “index of brain and cognitive health” (Figure 5).

Our construct of a BHI refers to a reliable index composed by a number of TMS-driven interactions ascertained repeatedly over time that would potentially assist in the evaluation of a given individual's level of brain and cognitive health across time (Figure 5). Building on an individual's natural BHI history might enable the detection of very early—potentially maladaptive—changes, thereby offering the opportunity of implementing individualized, preventive interventions. Furthermore, within the context of investigational trials aimed at preventing or delaying the onset of cognitive impairment and dementia, the BHI would not only provide an objective means for assessing trial's efficacy, but also, and importantly, a means to identify clinically normal individuals at highest risk for brain and cognitive decline on the basis of their BHI history, with potential repercussions for trial sample size.

While the establishment of a BHI will not be easy, we believe it is a most promising way to transform current conceptualizations of how to develop preventive and early interventions for neuropsychiatric disorders.

## Conclusions

Numerous lines of research suggest that brain and cognitive health are linked to preservation of brain plasticity and network dynamics, which underlie the brain's capacity to flexibly deal with challenges, acquire new skills, and adapt to change. We propose that neuronavigated, multimodal TMS measures are uniquely suited to offer serial assessments of an individual's brain plasticity and network dynamics across the lifespan, and integrated with cognitive, behavioral, genetic, and lifestyle factors may allow definition of a BHI. The ultimate goal is to track each individual's relative risk for cognitive decline, and thus develop and implement personalized interventions to prevent neuropsychiatric disorders.

### Conflict of interest statement

Dr. Freitas is co-inventor on a patent application on real-time integration of transcranial magnetic stimulation (TMS) with electroencephalography and functional magnetic resonance imaging (fMRI) for evaluating cortical plasticity impairments. Dr. Pascual-Leone serves on the scientific advisory boards for Nexstim, Neuronix, Starlab Neuroscience, Neosync, and Novavision, and is an inventor on patents and patent applications related to noninvasive brain stimulation and real-time integration of TMS with EEG and fMRI. Dr. Faranak Farzan declares that the research was conducted in the absence of any commercial or financial relationships that could be construed as a potential conflict of interest.

## Key Concept

Brain plasticity.: Brain plasticity can be conceptualized as nature's invention to overcome limitations of the genome and adapt to the rapidly changing environment. As such, plasticity represents an intrinsic property of the nervous system retained throughout life that enables modification of function and structure in response to environmental demands via the strengthening, weakening, pruning, or adding of synaptic connections and by promoting neurogenesis. This means that the brain does not remain static but, instead, continues to change as the obligatory consequence of each sensory input, motor act, association, reward signal, action plan, and awareness. Plasticity is essential to the establishment and maintenance of brain circuitry, it can be beneficial for the individual enabling acquisition of new skills and adaptation after an injury, but it can also account for the symptoms of disease.
Transcranial magnetic stimulation (TMS): TMS is a non-invasive procedure used to create electric currents in discrete brain regions. TMS is based on Faraday's principle of electromagnetic induction and features application of rapidly changing magnetic field pulses to the scalp via a copper wire coil connected to a magnetic stimulator. These brief pulsed magnetic fields painlessly pass through the skull and can create electric currents of sufficient magnitude in discrete brain regions to depolarize neurons. Trains of repeated TMS pulses (rTMS) at various stimulation frequencies and patterns can induce a lasting modification of activity in the targeted brain region, which can outlast the effects of the stimulation itself. Such lasting changes are thought to reflect alterations in brain plasticity mechanisms.
Brain Health Index (BHI): The BHI aims at integrating TMS-driven measures of brain plasticity in a thorough characterization of how the dynamics of local and network brain plasticity are, on one hand, able to sustain healthy functionality throughout life and how they may become impaired leading to neuropsychiatric diseases and disorders, and, on the other hand, how genetic (e.g., polymorphisms) and environmental factors (e.g., cognitive reserve), and their interactions, may influence the course of changes in the mechanisms of brain plasticity across the lifespan. This can be achieved by longitudinally assessing each individual's BHI. Furthermore, monitoring of each individual's BHIs throughout time would - theoretically, at least - enable the detection of very early, ‘outlier’, potentially pathologic changes prior to the clinical manifestation of brain and cognitive decline and disease. BHI monitoring during disease progression would further enable characterization of the pathophysiologic processes underlying the disease, which, in turn, could potentially expand the array of therapeutic targets. In addition, the BHI can act as a reliable index of treatment response. Ultimately, the BHI would enable the planning of individualized interventions to delay or remediate brain and cognitive dysfunction before clinical symptoms manifest and disability develops. The BHI may, thus, embody a promising way to transform current conceptualizations of how to develop preventive and early interventions for neuropsychiatric disorders.
